# Analysis of Urban Indian Organizations’ Promotion of Cancer Services

**DOI:** 10.1158/2767-9764.CRC-24-0335

**Published:** 2025-02-26

**Authors:** William O. Carson, Alyssa Little, Angela Monetathchi, Jennifer Erdrich, Felina M. Cordova-Marks

**Affiliations:** 1 Department of Health Promotion Sciences, Zuckerman College of Public Health, University of Arizona, Tucson, Arizona.; 2 University of Arizona, Tucson, Arizona.; 3 College of Medicine, University of Arizona, Tucson, Arizona.

## Abstract

The burden of cancer remains elevated for American Indians/Alaska Natives (AI/AN) in the United States, particularly in urban communities. Urban Indian Organizations (UIO) are a part of the Indian healthcare system for urban AI/AN populations to receive culturally competent care; therefore, it is important that UIOs convey the importance of cancer preventive and treatment options through their websites. The purpose of this study was to utilize the Indian Health Service (IHS) Office of Urban Indian Health Programs’ official website to identify, analyze, and describe the IHS-funded UIOs offering cancer-related services. We utilized qualitative content analysis on UIOs with primary care service capabilities. After determination of UIOs, each eligible website was reviewed to determine whether promotion of specific cancer-related services was being done based on the following categories: primary prevention, cancer screening, cancer support, and information/technology services that focus on the social determinants of health. There is limited, vague, or outdated information on UIO websites regarding the type of cancer-related services and programs provided. We found that whereas 21 of 34 UIOs list any mention of cancer screenings, few mentioned specific testing capabilities for various cancers. In addition, the listing of support and preventative services was also limited, with less than half of UIOs reporting on patient navigation services, among others. There is untapped potential in the utilization of websites for extending the impact of UIOs and their work with urban AI/AN populations in prevention, screening, and support of patients with cancer in a culturally appropriate manner that can improve outcomes.

**Significance:**

To the best of the knowledge of the authors, this is the first study to explore and measure cancer support services through public-facing channels. This study provides important data on how primary care facilities are engaging in public health promotion services in an online setting.

## Introduction

As of the 2010 U.S. Census, about 70% of American Indians/Alaska Natives (AI/AN) in the United States live within an urban setting (cited Jul 15, 2022, available at The American Indian and Alaska Native Population: 2010). AI/AN populations have seen consistent movement from rural and reservation lands to urban areas during the 20th century through termination of reservations, forced relocation, and other federal government policies (1). Urban Indian Organizations (UIO) are 501(c) (3) nonprofit organizations that are funded by the Indian Health Service (IHS) under the Indian Health Care Improvement Act and one of the main ways for AI/AN people to get access to culturally competent care in urban areas (2); however, it is important to note that not all UIOs are funded by the IHS. Despite serving a significant portion of the AI/AN population in the United States (3, 4), UIOs are expected to offer health services and care while only appropriated 1% of the total IHS budget (5).

Cancer disparities exist between urban AI/AN populations and urban non-Hispanic White populations and have increased over the last two decades; namely, for colorectal, cervical, and kidney cancers (6). AI/AN people in urban areas have increased mortality, decreased cancer screening usage, and less access to care for comorbidities associated with cancer, notably diabetes and chronic obstructive pulmonary disease (7). Primary care has a fundamental role in cancer prevention, as routine primary care check-ups are associated with increased cancer screenings (8) UIOs also provide other health services such as traditional healing, behavioral health, and community outreach to AI/AN people living in urban areas. Facilities may be fully ambulatory capable centers, limited ambulatory service, referral-based care, and finally, outpatient and residential alcohol and substance abuse treatment. (cited Jul 15, 2022, available at About Urban Indian Organizations | About Us).

AI/AN populations face barriers such as access to cancer screening services and treatment, as well as suboptimal education on different cancer types and on the importance of cancer screening and diagnostics (6, 9). Notable barriers to accessing cancer services for urban AI/AN populations include but are not limited to, e.g., the cost of treatment, coordinating care, transportation, and access to health information online. In a recent study, a majority of providers at UIOs cited out-of-pocket costs and lack of insurance as key reasons why patients were not getting dermatologic treatment (10). This tracts with other healthcare providers, and as a result of financial challenges, many healthcare providers/healthcare organizations offer insurance navigation services to get patients enrolled in eligible healthcare plans (cited Jul 15, 2022, available at Health Insurance Exchanges: Health Insurance “Navigators” and In-Person Assistance). Additional barriers to care include difficulty scheduling visits and limited access to public transportation to reach appointments (9, 11). Today, patient navigation services are now widely implemented through IHS clinics to assist in the coordination of complex treatment plans (12, 13).

Limited research exists around individuals seeking health information online, with prior studies examining whether healthcare clinics provide sufficient information for their patients on their websites with mixed results (14). The U.S. Census Bureau tracks internet access and usage on tribal lands (cited Jun 25, 2024, available at Broadband Access in Tribal Areas Lags Rest of the Nation) but not for urban areas. In addition, research on the health seeking behaviors of AI/AN populations are also limited but exists. In a 2011 study, 48% of AI/AN participants utilized the internet to access health information (15). In a 2014 study, researchers found that AI/AN populations vary in seeking health information online depending on age group, with younger people being more willing to seek out and trust online sources and the older generations being more hesitant (16). Unfortunately, there is little in the way of health-seeking behavior data online for AI/AN populations since the rise of popularity of smart phones and social media.

Cancer screening services, such as colorectal cancer screening, have been advantageous in preventing mortality and decreasing the incidence of cancer (17). Currently, there are no studies published that examine the cancer-related services offered by UIOs and no studies examining the way information is presented to the public through the websites of UIOs. The purpose of this study is to utilize the IHS website to identify the UIOs that urban AI/AN populations have access to and describe what cancer-related resources (prevention, screening, treatment, and support services) each UIO provides patients according to their public-facing websites.

## Materials and Methods

**Figure 1:**
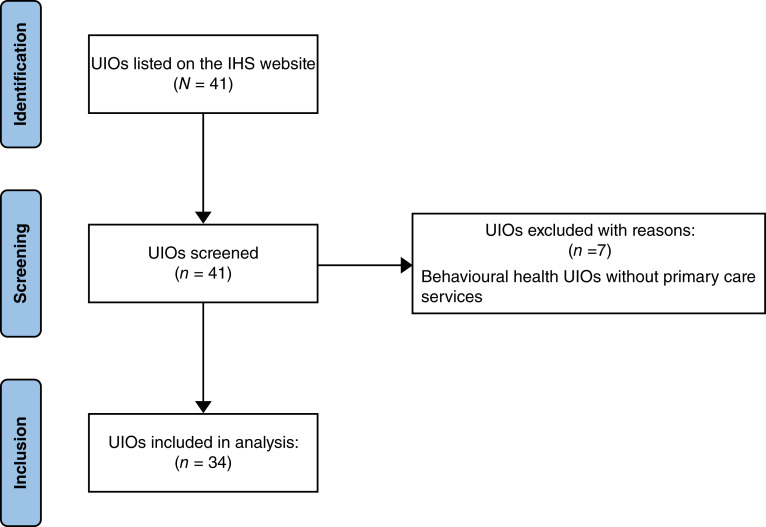
Inclusion and exclusion criteria for the determination of UIOs’ status as primary care providers.

In June 2022, the research team began a qualitative observational review of the UIOs listed on the IHS website utilizing content analysis (cited Jul 15, 2022, available at Urban Indian Organizations | Office of Urban Indian Health Programs). According to the IHS website, a total of 41 UIOs are listed. Utilizing this initial list, three reviewers conducted a prescreening to determine which UIOs offered primary care services. To be included in this study, UIOs had to offer primary care services. This screening resulted in 34 UIOs making the final list as 7 of the UIOs only offer behavioral health, thus making them ineligible to be included, seen in Fig. 1. Further information on the list of UIOs included is in Supplementary Table S1. To ensure that the findings were accurate, the research team remained in contact with the IHS Office of Urban Indian Health Program for consultation regarding data accuracy and review of multiple manuscript drafts prior to submission for publication.

Prior to the start of the review, a team of three reviewers established definitions for how and why certain services in the categories of primary prevention, cancer screening, support services, and information technology services were determined. Eligible UIOs’ websites were then analyzed according to the preset review guidelines. Data were then extracted for each specific point and recorded on a shared team file.

### Primary prevention criterion

The preventive programs defined in our study are services focusing on wellness. They were examined to see whether UIOs advertised health programming. Wellness included, but was not limited to, prevention programs, such as traditional food programs and diabetes management programs. Diabetes was included as it may be a risk factor for certain types of cancer such as colorectal cancer (18).

### Cancer screening service criterion

Screening services included both laboratory-based tests and medical imaging. In addition to general cancer screening availability, sites particularly mentioning screening for predominant cancers such as breast, colon, prostate, cervical, and lung cancers were examined. Review criteria included the following cancer screening tests as defined by the American Cancer Society (ACS): (i) cervical cancer screening which can include a human papillomavirus test as well as a pap smear for abnormal cells (cited Jun 25, 2024, available at The American Cancer Society Guidelines for the Prevention and Early Detection of Cervical Cancer | American Cancer Society); (ii) prostate cancer screening which utilizes the prostate-specific antigen (PSA) blood test, (iii) mammograms which are used for screening breast cancer; (iv) lung cancer screening which entails low-dose CT scans, and (v) colorectal cancer screening which can include fecal-based tests and colonoscopy (cited Jun 25, 2024, available at Colorectal Cancer Screening Tests | Sigmoidoscopy & Colonoscopy | American Cancer Society). Based on the ACS list of cancer screening, we then reviewed UIO websites for the following six ACS-identified cancer screening tests: fecal tests, colonoscopies, PSA test, human papillomavirus test, CT chest scans, pap smears, and mammograms.

### Cancer support service criterion

UIO cancer support services included patient navigation, insurance navigation, transportation to and from the clinic, availability of payment sliding scales based on income, and other forms of financial assistance programs, which can assist with financial hardships that many cancer survivors report come with treatment (19, 20).

### Information and technology criterion

The last section to be evaluated was whether UIO websites could be confirmed as updated. This was determined by either publication dates on the websites or by examining what year was mentioned in the copyright date. If the reviewers could not identify a date, then “not listed” status was marked for the date.

### Data availability

Data were generated by the authors and are available on request.

### UIO website review process

When a program or medical test was identified on a UIO website, the research team marked this clinic with the phrase, “yes,” “referral,” or “not listed.” A UIO listed as “referral” indicated that although they did not conduct the tests on-site, they would refer their patients to another clinic. A UIO “not listed” indicated that no program or test was identified on their website.

Content analysis was limited to UIOs listed on the IHS Office of Urban Indian Health Programs. This website lists 41 UIOs, and after screening for only those offering healthcare services, the number was reduced to 34 after verifying the data with Urban IHS National Leadership. Thirty-four UIO websites self-reported availability of cancer preventive, cancer screening, and cancer support services and are displayed in Table 1.

**Table 1:** Findings of the 34 UIOs reviewed for wellness programs, cancer screening services, cancer support services, and information/technology support

*N* = 34	Yes % (*n*)	Referral % (*n*)	Not listed % (*n*)
Wellness programs			
Traditional food program	29.4 (10)	0 (0)	70.6 (24)
Diabetes management program	88.2 (30)	0 (0)	11.8 (4)
Cancer screening services			
Cancer screenings (any mention)	61.8 (21)	17.6 (6)	20.6 (7)
Colorectal cancer screenings (fecal tests, colonoscopies, etc.)	0 (0)	23.5 (8)	76.5 (26)
Lung cancer screening (Low-dose computed tomography)	0 (0)	17.6 (6)	82.3 (28)
Prostate cancer screening (PSA test)	0 (0)	17.6 (6)	82.3 (28)
Cervical cancer screening (pap smears)	20.6 (7)	17.6 (6)	61.8 (21)
Human papillomavirus test	5.9 (2)	17.6 (6)	76.5 (26)
Breast cancer screening (mammography)	32.3 (11)	50 (17)	17.6 (6)
Laboratory and X-ray services	29.4 (10)	50 (17)	20.6 (7)
Cancer support services			
Financial assistance program	38.2 (13)	0 (0)	61.8 (21)
Payment sliding scale	44.1 (15)	0 (0)	55.9 (19)
Patient navigation	44.1 (15)	0 (0)	11.8 (4)
Insurance navigation	44.1 (15)	0 (0)	55.9 (19)
Transportation	29.4 (10)	0 (0)	70.6 (24)
Information/technology support			
Webmaster listed	17.6 (6)	N/A	82.3 (28)
Social media links	94.1 (32)	N/A	5.9 (2)
Updated website in 2022	55.9 (19)	N/A	44.1 (15)

To ensure that the results were internally accurate, the primary reviewer checked each website for the listed categories in two separate rounds. In each round, the reviewer would exhaustively attempt to identify whether the specific UIO made any mention to having the service under review. A secondary reviewer fact-checked the results for a total of three unique reviews for every UIO website.

Reviewers then met to discuss any discrepancies in findings and resolved these with the assistance of senior researchers on the team. The results were logged in Excel after each review, with the final information then calculated in a pivot table. The final totals for each variable were then entered and displayed in Table 1. At the conclusion of the review process by the research team, we then consulted with the IHS Office of Urban Indian Health Programs to corroborate the accuracy of our data.

## Results

### Prevention

Among the 34 UIOs which provide healthcare services, almost a third, 29.3% (*n* = 10), of the organizations described programs which offered traditional food services for their patients. The vast majority, 88.2% (*n* = 30), of UIOs promoted diabetes management programs as core services provided to clients.

### Screening

Of the 34 UIOs, 61.8% (*n* = 21) reported on their websites that they offer cancer screening services, with another 17.6% (*n* = 6) offering referrals to outside organizations. When looking for the names of the prior-identified cancer screening tests as specified by the ACS, the results were mixed, with some organizations listing specific cancer screenings available at the clinic whereas others listed a broader, more general description of “screenings.”

Breast cancer screenings (or the term “mammography”) were the most reported specific cancer screening test that UIOs offer, with 32.4% (*n* = 11) describing that patients can be screened with a mammogram at their clinic, and another 17 websites offering referrals. Pap smears (cervical cancer screening) were listed by 20.6% (*n* = 7) of organizations as a test offered by their clinics; 0% (*n* = 0) of clinics listed colorectal cancer screening services on-site, with two clinics, 5.9% (*n* = 2), advertising referrals. Likewise, 0% (*n* = 0) of the organization websites described their capability to conduct PSA tests used for prostate cancer screening. Less than half of all clinic websites mentioned laboratory and X-ray services; 29.4% (*n* = 10) offered these services at their site, with another 50% (*n* = 17) providing referrals to other facilities.

### Support services

For patient navigation services, 44.1% (*n* = 15) of the sites had these listed, and of the UIOs reviewed, 44.1% (*n* = 15) listed insurance navigation as a specific service that is provided by their organization. Financial assistance programs are mentioned by 38.2% (*n* = 13) of the UIOs, with 44.1% (*n* = 15) of the UIOs analyzed specifically mentioning the availability of a payment sliding scale. Finally, 29.3% (*n* = 10) of the organizations listed transportation services for their clients.

### Information/technology

Finally, we examined whether UIOs had listed a designated staff member to assist in the maintenance of the website and whether the website had been updated since January 1, 2022. Only 17.6% (*n* = 6) of the UIOs mentioned a webmaster on their organization pages. According to our review, 55.9% (*n* = 19) of the UIO websites had been updated since the start of 2022.

## Discussion

AI/AN populations living in urban areas rely on UIOs to receive culturally competent care when they do not have access to the resources offered through the IHS or tribally operated healthcare facilities on tribal lands (cited July 15, 2022, available at Urban Indian Health Program Fact Sheet). Despite funding limitations, UIOs continue to provide care for the large population of urban AI/AN people who may otherwise be challenged with finding medical services. This review has demonstrated that all 34 UIOs examined provide health-related services, such as cancer prevention and cancer support services, based on their public-facing websites. However, it is evident that each website’s breadth of information available to patients varied, such that some program websites demonstrated more detailed, updated information compared with others. UIOs seem to provide cancer programming, such as cancer screenings and referrals, when necessary, for AI/AN populations in their urban areas but do not consistently list these services or update information on their webpages. This review highlights a potential need to update websites for better descriptions of the specific healthcare services offered at each clinic.

### Improving access to information around cancer services

Early detection and treatment can lead to a more positive prognosis for cancer. It is essential to provide patients with adequate information on what cancer screenings are necessary for an individual in the specific age range to improve health literacy and encourage early and frequent testing. The majority of UIOs did not mention the types of cancer that are readily screened on-site, and which types of cancer screening would need to be referred to a medical facility that has the necessary medical equipment for screening.

Health communication through online websites and social media is an effective way to communicate with urban AI/AN populations. Previous research found that an urban AI/AN population reported 98% access to computers, 97% access to emails, and 94% access to mobile phones (21). With this high percentage having access to technology, efforts to increase clarity and centralization of online sources for UIO services, including cancer screening services and other supportive programming, could potentially lead to better patient outcomes. The AI/AN population is a younger population, with 27.5% of the population being under the age of 18 compared with 18.6% of the Non-Hispanic White people as of 2019 (cited Jun 25, 2024, available at Demographics | NCAI). Younger generations are more familiar with current technology and can utilize the internet, which makes it more important to modernize and update UIO websites and databases on national program websites. In addition, the youth/younger family members may provide caregiving for an elder family member that includes finding healthcare information for the care recipient (22). In addition, urban AI/AN populations are more diverse in comparison with AI/AN populations on reservations, with urban communities representing dozens, if not hundreds, of tribes (23). In recent research, as many as 50 tribal nations are represented in urban high schools in Seattle (23). Leading urban AI/AN health organizations should collaborate to streamline electronic health information on cancer screening, treatment, and public health education for ease of use in the modern, digital urban space.

The majority of UIOs did not list staff members in charge of their websites, and almost half did not update their sites within the past year. This is a potential area that can be addressed with additional funding and support mechanisms from federal and national agencies. The results of this project demonstrate the potential for UIOs to increase their impact on cancer care, treatment, and education through enhancements to their online presence.

### Suggestions for the IHS and UIOs

Increased funding for webmaster, social media, and health education positions may be necessary for UIOs to regularly update their websites and maintain a steady online presence with their patient community. Considering the importance of cancer screening and the impact of cancer on the AI/AN population, it may be beneficial to improve the level of information that patients can receive from their UIOs on websites. There does not seem to be uniformity in what general health information is presented on UIO websites. Few UIOs mention having a webmaster or social media employee which may help explain why websites are so varied in available information regarding cancer screening, treatment, and referral programs available. There may be practical concerns that limit the specificity of online information, such as varying budgets and resources that impact the consistency of service availability, or employee turnover that limits named points of contact, but this is why increased funding for updating the sites reflective of these dynamic variables should be a priority so that UIOs have an accurate online presence. Additional work is needed to improve public health messaging on organization websites, and it should be examined whether this is an area in which additional funding through budget increases from the IHS is needed to fully staff critical positions that affect patients.

The limited information on many of the UIO websites may dissuade prospective and current patients from utilizing the websites and understanding what types of services the organizations provide. One such service that can address this concern is patient navigators, which have been beneficial in improving cancer screening outcomes, including colorectal cancer screening, as well as improving patient satisfaction and trust in healthcare services in a culturally responsive manner (24). Whether improvements come from the IHS, the Centers for Disease Control and Prevention, the ACS, the Urban Indian Health Institute (UIHI), or other organizations, clinic websites should offer direct information or link to outside reputable sources of information on the importance of cancer prevention, screenings, and support services.

### Limitations

We have examined the ways in which UIOs present their services to the public through their healthcare websites; however, it is important to recognize that we cannot say that if a program is not listed, it is not offered. We can only report that the program is potentially unavailable at the UIO or is unadvertised. This is also why in the findings, we state that a program is “not listed” as opposed to “not available.”

One of the strengths of this research, which is the attempt to truly examine how prospective AI/AN patients may see how UIOs promote their services, is also a limitation. As seen in Table 1, this study cannot confirm whether certain programs and services are not offered by the UIOs. Instead, all that can be said is that it is not clear based on the website, as of July 2022. Another challenge as stated in the prior section, “Suggestions for the IHS and UIOs,” is that UIOs are not the only form of care for urban AI/AN populations. In fact, prior research supported by the Centers for Disease Control and Urban Indian Health Institute states that there are 72 total health centers supplying care to urban AI/AN populations (6). This study only includes organizations directly funded by the IHS. We acknowledge that this can paint an incomplete picture of the challenges faced by AI/AN communities. It is important that future research includes as many of the 72 total centers in future evaluations.

This led to another issue that came up during this review. We chose to use only UIOs funded by the IHS as initial attempts to understand how many urban area health organizations that care for AI/AN populations were unsuccessful. There are several organizations which fund urban AI/AN health programs, and all maintain their own lists, thus it is challenging to quickly identify health programs available in each urban area. The IHS Office of Urban Indian Health Programs lists 41 organizations that are IHS-funded within the 12 IHS service areas. Analogously, the UIHI lists 46 urban health organizations they support which assist AI/AN populations on their website, with several cities and regions listed that the IHS does not include on their website. In addition to the IHS and UIHI, the Centers for Disease Control and Prevention and tribal organizations fund UIOs and other organizations assisting urban AI/AN people. Leading organizations should collaborate to provide exhaustive lists on which cancer care services are provided in every metro area for urban AI/AN populations. The current dispersed nature of information for urban AI/AN may negatively impact patient outcomes (6).

These limitations being stated, the research shows a clear need for UIOs to better promote the range of cancer services offered at their clinics. This is a novel approach to examining how cancer service promotion and communication can be utilized to improve patient education related to cancer care.

### Conclusion

UIOs offer a wide range of cultural, medical, and public health services to the urban AI/AN population. A review of these websites highlights potential areas for improvement that can benefit patients, leading to more positive health outcomes. UIOs should examine how services should be described on their websites, which educational materials are made available/which should be added, and a website management plan that can ensure the most up-to-date information is presented.

## Supplementary Material

Supplementary Data List of UIOs analyzed for this review
