# Supplementary material for: Analysis of Urban Indian Organizations’ Promotion of Cancer Services
Source: Cancer Res Commun. 2025 Feb 26;5(2):369–74. doi: 10.1158/2767-9764.CRC-24-0335 (PMC11863186; doi:10.1158/2767-9764.CRC-24-0335)
Supplement: Supplementary Data — List of UIOs analyzed for this review [file crc-24-0335_supplementary_data_suppsd.pdf]

Urban Indian Organization
Denver Indian Health & Family Services, Inc.
First Nations Community HealthSource
American Indian Health Service of Chicago, Inc.
Gerald L. Ignance Indian Health Center, Inc.
American Indian Health & Family Services
Indian Family Health Clinic
Missoula Urban Indian Health Center
Helena Indian Alliance - Leo Pocha Clinic
Friendship House - Association of American Indian, Inc. of San Francisco
Bakersfield American Indian Health Project
Fresno American Indian Health Project
American Indian Health & Services, Inc.
Indian Health Center of Santa Clara Valley
Native American Health Center
San Diego American Indian Health Center
Sacramento Native American Health Center, Inc.
United American Indian Involvement, Inc.
Nebraska Urban Indian Health Coalition, Inc.
South Dakota Urban Indian Health
Native American Lifelines of Baltimore and Boston
New York Indian Council, Inc.
Native Americans for Community Action, Inc.
Urban Inter-Tribal Center of Texas
Kansas City Indian Center
Hunter Health
Indian Health Care Resource Center of Tulsa
Oklahoma City Indian Clinic
Native Health
Nevada Urban Indians, Inc.
Urban Indian Center of Salt Lake
Native American Connections
The NATIVE Project
Seattle Indian Health Board
Tucson Indian Center
